# Scaling of damage mechanism for additively manufactured alloys at very high cycle fatigue

**DOI:** 10.1038/s41598-024-61033-2

**Published:** 2024-05-13

**Authors:** B. S. Voloskov, M. V. Bannikov, Y. V. Bayandin, O. B. Naimark, I. V. Sergeichev

**Affiliations:** 1 Center for Materials Technologies, Skolkovo Institute of Science and Technology, Moscow, 121205 Russia; 2 Institute of Continuous Media Mechanics UB RAS, Perm, 614013 Russia

**Keywords:** Additive manufacturing, Very high cycle fatigue (VHCF), High cycle fatigue (HCF), Fine granular area (FGA), Multifractal analysis, Condensed-matter physics, Mechanical properties, Metals and alloys

## Abstract

A comparative analysis of fracture mechanisms in high- and very high- cycle fatigue (HCF, VHCF) regimes was carried out based on the results of multifractal analysis of the fracture surfaces of additively manufactured 316L stainless steel samples. In terms of scale invariants, the morphology of fracture surfaces in HCF and VHCF regimes inside and outside the fine granular area is shown. The analysis demonstrated that chaotic patterns of relief formation prevail in the crack initiation zone of VHCF samples. However, there is a self-similar relief with a pronounced correlation in the crack propagation area. The relief of the crack growth areas for HCF and VHCF samples are similar to each other.

## Introduction

Additive manufacturing (AM) or so-called 3D printing is a rapidly growing technology that involves the use of various methods to build parts from a digital design^1^. This technology has been increasingly used in a wide range of industries, including aerospace, automotive, medical, and industrial manufacturing. AM metals and alloys can be used for applications in which parts can be subjected to high cyclic fatigue and very high cycling fatigue. Additive Manufacturing offers considerable potential for producing metal components. However, there are some associated limitations to bear in mind. These include the presence of defects, such as a lack of fusion (LoF), voids, mechanical anisotropy^2^, the high cost of raw materials used in the process. In addition, parts built through the AM process are prone to high residual stress (RS)^3^, due to the variable thermal history resulting from rapid melting and cooling. RS stimulates microcracks in the material^2^. Most significantly, these parts have often low fatigue strength because of the high surface roughness and presence of defects such as porosity and LoF^4^. However, different post treatments, for instance, hot isostatic pressing (HIP)^5^ or post laser polishing^6^can improve the fatigue endurance of AM metals.

316L is a type of austenitic stainless steel that is well-known in the marine, aerospace, chemical, petroleum, and medical industries^7^. This stainless steel is renowned for its thermal stability, corrosion resistance, and ability to maintain its toughness even in extremely low temperatures. It also shows a low susceptibility to hydrogen embrittlement while exposed to a hydrogen rich setting, which makes it ideal for certain industrial applications. Moreover, this steel has become a popular choice for additive manufacturing processes due to its excellent weldability and general versatility^3^. However, despite the extensive research on the fatigue characteristics of AM 316L stainless steel, there is a dearth of experimental information that is required to comprehend VHCF behavior^8,9^. The industrial use of AM 316L would be expanded with the help of such knowledge.

A quantitative analysis of the relief of the fracture surface in terms of universal statistical scale characteristics makes it possible to establish a correlation between the measured roughness and the macromechanical characteristics of the material^10^. The development of methods for the fractal analysis of fracture surfaces began with the work^11^, where a method was proposed for determining the fractal dimension and the relationship between this characteristic and the impact strength of metals.

A discussion of the results of studies of the damage development mechanisms in the VHCF regime is presented in^12^. The formation of the FGA region in the VHCF regime is initiated for materials by microscopic localization of plastic deformation, which, in turn, is caused by the anisotropic elastic behavior of grains, the discrete nature of slip systems, the shape of grains, and their crystallographic orientation^13,14^. Thus, a fatigue crack is initiated in the volume of a defect-free material for multiphase steels^15,16^ . It was shown by Chai^17^ that the process of crack initiation happens because of cyclic plastic deformation of a soft phase, for example, a ferritic phase due to rupture of deformations of adjacent phases.

The fine granular area (FGA) which originates as a consequence of VHCF loading can be observed in the vicinity of the crack-initiating defect. The precise origin of the FGA is not fully comprehended and there is not a consensus about what mechanism describes its formation. Additionally, there is no single point of view regarding if the FGA is a prerequisite or an aftereffect of the crack initiation period^18^. Nevertheless, it has been proven that the formation of FGA takes up more than 90%^12^ or even 95%^19^of the fatigue life. Thus, it is important to comprehend the mechanisms of FGA formation in order to understand the material's behavior in VHCF. However, the possible models of FGA formation exist. Sakai proposed a model of crack initiation in VHCF with three stages^20^. The first stage is marked by intensive polygonization around an interior defect, leading to the formation of a FGA. The second stage involves nucleation and coalescence of micro-debondings. The third stage is the spread of micro-debondings over the FGA, forming a penny-shaped crack around the interior defect. The model by Murakami et al.^21^ implies hydrogen embrittlement coupled with a cyclic loading. Another mechanism is dispersive decohesion of spherical carbide proposed by Shiozawa et al.^22^. There are other models suggested by other researchers^23–25^. A phenomenological relationship was suggested^26,27^ to describe the crack growth kinetics for sizes smaller than the "Paris cracks". This relationship, considering the macroscopic characteristics of the stress state at the crack tip, incorporated the structural parameters of the Burgers vector and the effective stress intensity factor. Naimark et al. found^28^ that the distinct stages of VHCF damage-failure transition are associated with different qualitative mechanisms of crack initiation, crack growth, and crack advance, which are linked to the nonlinearity of the free energy release.

Unlike traditional metals and alloys, additively manufactured materials are characterized by a variety of internal defects—pores, non-metallic inclusions, LoF defects, unmelted particles, which can initiate the formation of FGA^8,29^ and the subsequent development of a fatigue crack. The comprehensive review of the VHCF of AM materials is published by Caivano et al.^30^. The authors provided the summary of the data on the VHCF response of various materials including aluminum alloys, Inconel 718, Ti-6Al-4 V, and 316L, which were produced through different additive manufacturing techniques.

Scanning electron microscopy (SEM) analysis of the fracture surfaces of AM 316L samples subjected to HCF and VHCF loading showed that the morphologies of fracture surfaces are similar in these two regimes outside the FGA region^8^ for two types of defects.

The identity of fracture surfaces corresponding to different types of defects and loading regimes, and, consequently, the mechanism of fatigue crack development, can be established based on a comparison of the scale invariants of these surfaces relief. Scale invariants of the fracture surface topography can be obtained using multifractal analysis^11^. A review of fractal scaling models in fracture mechanics is presented in^31^. In the last decade, this method has been developed in^32–34^.

## Experimental procedures, methods and materials

The sample manufacturing and experimental procedures were described previously^8^. However, the key points are described below. The cylindrical bars were vertically manufactured by LPBF using a TruPrint 1000 machine by Trumpf (Ditzingen, Germany). The focus spot diameter of the laser beam is 55 µm and it has a Gaussian profile. The samples were printed using “chessboard” scanning strategy with printing parameters shown in Table 1. “Chessboard” strategy is the strategy where the layer is equally divided by squares. The scanning directions between these squares are always mutually perpendicular. The angle between layers is changed consistently by an angle of 90°. The dimensions of the squares were with the length of side equal to 250 µm.

**Table 1:** Main manufacturing parameters.

Laser power [W]	Beam traverse speed [mm/sec]	Hatch distance [mm]	Layer thickness [mm]
113	700	0.08	0.020

The geometries and dimensions of the printed bars and test samples that were milled from the cylinders by the CNC machine shown in [8]. The static tensile test samples were machined to have the same shape as conventional fatigue samples. No samples underwent thermal treatment.

The conventional fatigue tests were carried out using an Instron 8801 (Norwood, MA, USA) servo-hydraulic machine with cycle asymmetry coefficient R = 0.1 (R = σ_min_/σ_max_) at a frequency of 30 Hz and at room temperature.

Quattro S microscope by ThermoFisherScientific (Netherlands) was used to conduct the SEM investigation.

The axial tension–compression VHCF tests were performed on a Shimadzu USF-2000 ultrasonic testing device (Kyoto, Japan). The external frequency supplied by the test machine must be the one of the natural frequencies of the sample. This fact defines the geometry of the sample for ultrasonic testing. The sample is heated by repeated loading at high stress and high speed. Therefore, forced air cooling and a periodically operating drive cool it. A periodically operating drive involves repetition of the cycle so that ultrasonic waves are created in a short time and then switched off for a while. The samples were put through the ultrasonic test that involved a 300 ms pulse and a 500 ms pause for additional cooling. The displacement of the free end of the sample was gauged using an eddy current extensometer. When the resonance frequency varied by more than 500 Hz from the initial setup frequency, the tests were automatically terminated meaning that the sample was damaged. The damaged samples were loaded to (brittle) failure freezing in liquid nitrogen to reveal the fracture surface and image its morphology after the test^8^.

The axial tension–compression VHCF tests were performed on a Shimadzu USF-2000 ultrasonic testing device (Kyoto, Japan). The external frequency supplied by the test machine must be the one of the natural frequencies of the sample. This fact defines the geometry of the sample for ultrasonic testing. The sample is heated by repeated loading at high stress and high speed. Therefore, forced air cooling and a periodically operating drive cool it. A periodically operating drive involves repetition of the cycle so that ultrasonic waves are created in a short time and then switched off for a while. The samples were put through the ultrasonic test that involved a 300 ms pulse and a 500 ms pause for additional cooling. The displacement of the free end of the sample was gauged using an eddy current extensometer. When the resonance frequency varied by more than 500 Hz from the initial setup frequency, the tests were automatically terminated meaning that the sample was damaged. The damaged samples were loaded to (brittle) failure freezing in liquid nitrogen to reveal the fracture surface and image its morphology after the test8.

The MF-DFA algorithm (multifractal method based on the analysis of fluctuations of the analyzed data with excluded slope) was used to construct the multifractal spectrum of morphology of fractured surface profiles. This method is widely used in the studies of temporal and spatial series of different nature^35,36^. Unlike the methods using fast Fourier transforms and wavelet transforms, the MF-DFA method^37^ uses the direct method of constructing partial functions *Z(q,s).* Partial functions for different values of *q* are calculated as follows.

1 $$Z\left( {q,s} \right) = \left\{ {\begin{array}{*{20}c} {\left\{ {\frac{1}{{N_{s} }}\mathop \sum \limits_{\nu = 1}^{{N_{s} }} \left[ {F^{2} \left( {\nu ,s} \right)} \right]^{q/2} } \right\}^{\frac{1}{q}} ,q \ne 0} \\ {\exp \left\{ {\frac{1}{{2N_{s} }}\mathop \sum \limits_{\nu = 1}^{{N_{s} }} \ln \left[ {F^{2} \left( {\nu ,s} \right)} \right]} \right\},q = 0} \\ \end{array} } \right.$$

where ${F}^{2}\left(\nu ,s\right)$ are the standard deviations of the function studied from the linear trend on the *v*-the interval of length *s*.

In expression (1), *q* is the degree reflecting the influence of the contribution of large-scale fluctuations, at *q* > *0*, and small-scale fluctuations, at *q* < *0*^38^. By the obtained partial functions $Z\left(q,s\right)$, where *s* plays the role of scale, in the range ${s}_{min}\le s\le {s}_{max}$, in which the degree dependence $Z\left(q,s\right) {s}^{\alpha \left(q\right)}$ is valid, the generalized indices *α(q)* are constructed.

The multifractal spectrum (spectrum of singularities) *D(H)* is determined using the Lejandre transformation

2 $$\left\{\begin{array}{c}H=\alpha \left(q\right)+\frac{q\partial \alpha \left(q\right)}{\partial q}\\ D\left(H\right)={q}^{2}\frac{\partial \alpha \left(q\right)}{\partial q}+1\end{array}\right.,$$

The spectrum width $\Delta H=\left|H\left(q=-\infty \right)-H\left(q=+\infty \right)\right|$ determines the degree of multifractality of the investigated signal, and the characteristic values of the singularity spectrum function *D(H)* and its derivative at various positive values *q* = 0,1,2, etc. characterize the corresponding fractal dimensions^38^. At *q* = 0 the maximum value of the function $D\left({H}_{max}\right)={D}_{0}=1$ is reached, where ${H}_{max}={H}_{q=0}$, which coincides with the Hausdorff fractal dimension and is the dimension of the embedding space—the domain of the analyzed function.

## Results

**Figure 1:**
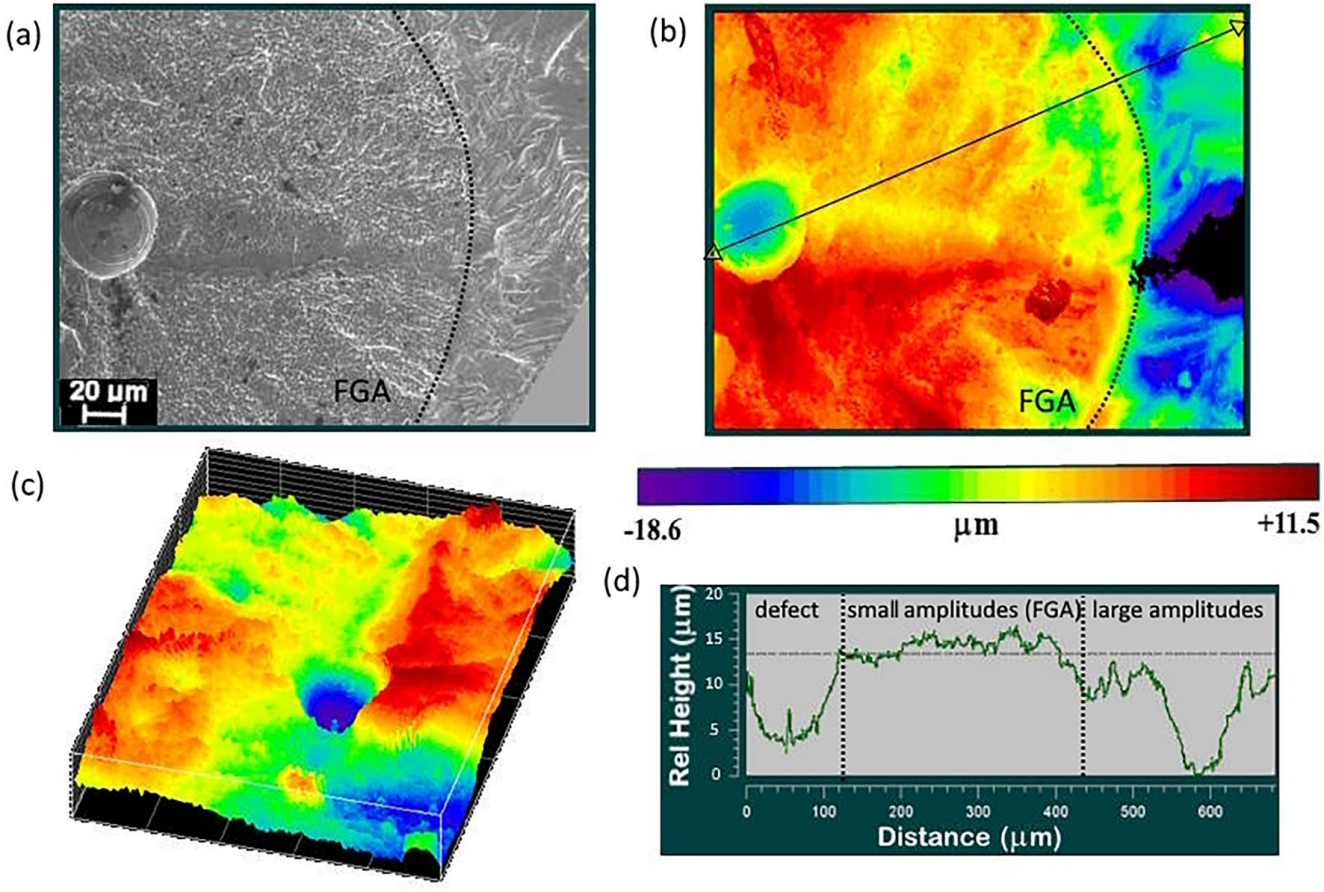
The surface relief of the fractured sample after VHCF loading: (**a**) Fracture surface image obtained by SEM; (**b**) Fracture surface image obtained on the profilometer-interferometer NewView (top view); (**c**) Fracture surface image obtained on the profilometer-interferometer NewView (isometric view) (**d**) One-dimensional profile of the surface.

The surface relief of the fractured samples was observed using the high-resolution interferometer profilometer NewView (Fig. 1). Then analyzed by fractal analysis methods (MF-DFA) to determine the conditions of correlated behavior of multiscale defective structures, which was associated with the origin and propagation of the fatigue crack.

The surface profile in the area of crack origin has significant differences with the area of further crack growth (Fig. 1d). In the area of fatigue crack formation, frequent fluctuations of the profile with a small amplitude are visible, which may correspond to the formation of a subgrain or fine granular area. In the crack growth region, smoother profile fluctuations are visible, but with larger amplitudes, which correspond to the germination of the fatigue crack to certain lengths.

**Figure 2:**
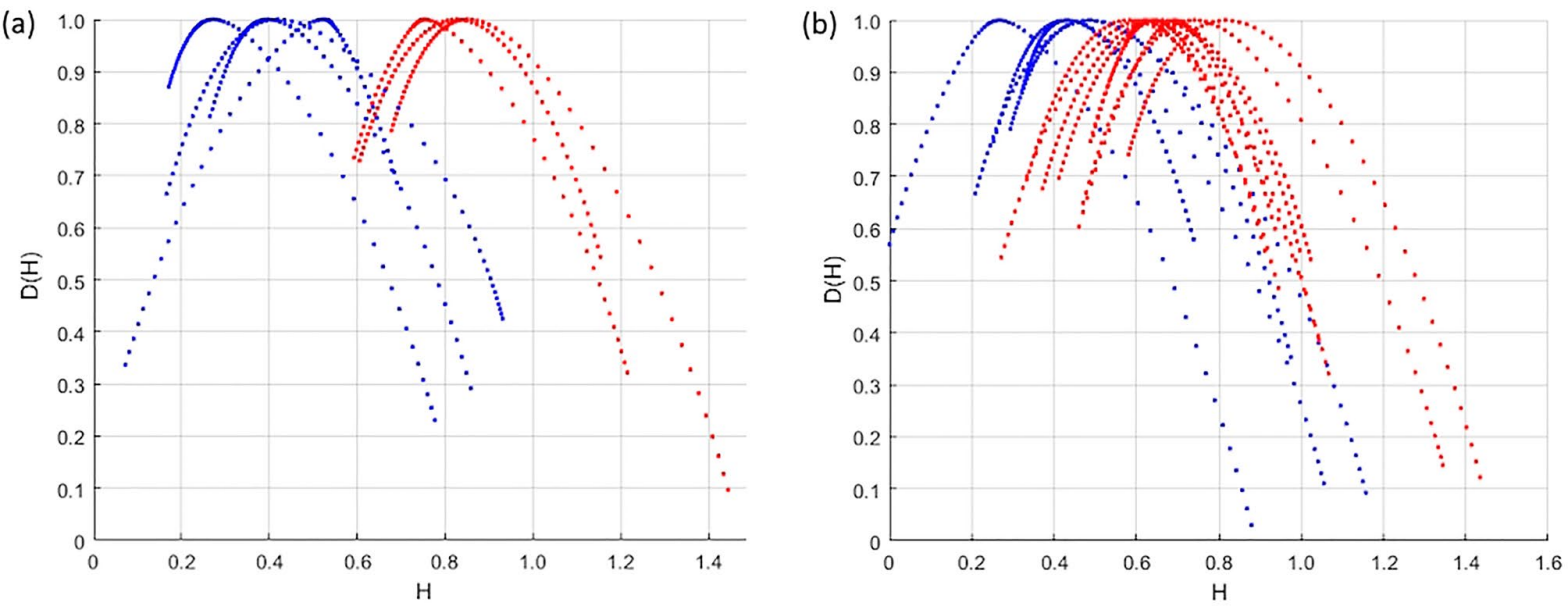
A characteristic view of the dependence of the fractal dimension spectrum – D(H) on the Hurst index—H: blue in the area of crack origin, red in the area of crack growth (**a**) Magnification × 500 (**b**) Magnification × 2000.

**Figure 3:**
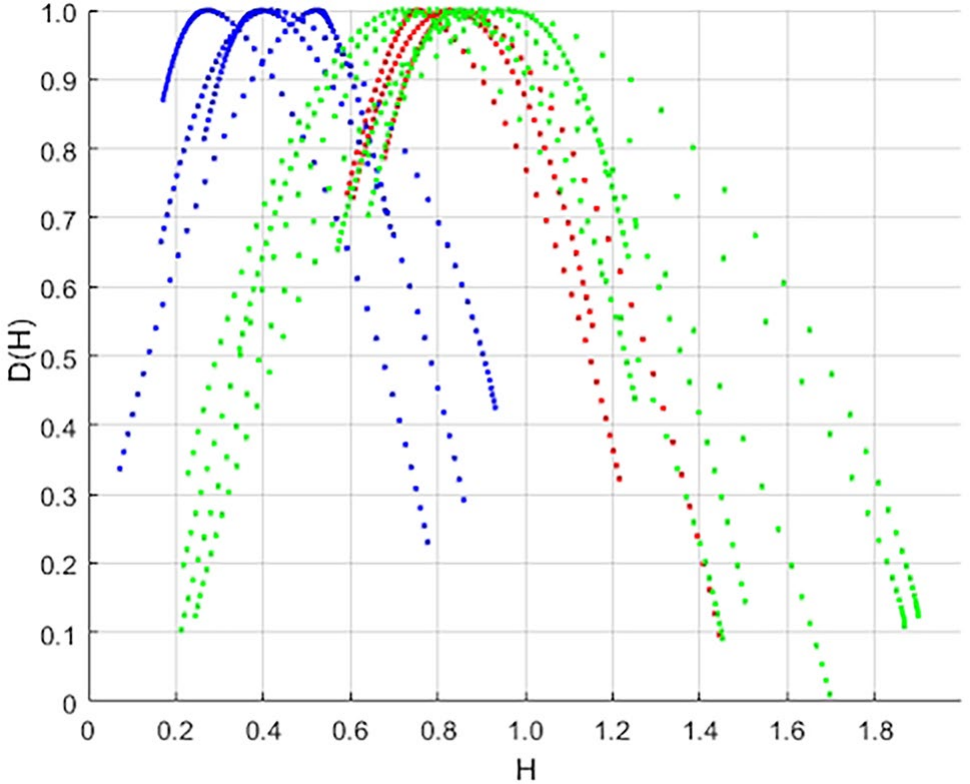
A characteristic view of the dependence of the fractal dimension spectrum – D(H) on the Hurst index—H: blue in the crack nucleation region, red in the crack growth region, green—a sample destroyed in the HCF regime with a surface crack.

The sets of spectra constructed by the MF-DFA method from profiles inside the crack nucleation region and its propagation region are shown in Fig. 2 and Fig. 3. The spectra plotted inside the FGA regions are blue, and in the fatigue crack propagation regions – red. In Fig. 3, the spectra obtained from samples fractured by high cycle fatigue without the formation of the FGA are plotted in green.

According to the graphs, it can be concluded that chaotic patterns of relief formation prevail in the crack origin zone (Hurst index 0.25–0.5), and there is a self-similar relief with pronounced correlation in the crack propagation area (Hurst index 0.75–1). As can be seen from Fig. 3, regularities, the spectra obtained on slices in the crack growth areas for HCF and for VHCF samples are comparable to each other. However, spectra are slightly wider in terms of the values of the H index for samples without FGA.

The quantitative differences in the roughness-scaling can be linked to the criticality stages for damage-failure transition established by Naimark et al.^28^. The crack origin area with narrow D(H) spectrum for VHCF regime is related to the specific self-similar “blow-up” solution of damage localization kinetics over characteristic length (crack origin zone). The propagation of this area depends strongly on damage kinetics in FGA area due to coherent kinetics of localized slips, following grain fragmentation up to the stage of transformation of this area into the secondary damage localization zone. The formation of FGA occurs over numerous cycles due to the intense polygonization around the interior defect^39^. The second stage is characterized by the nucleation of micro-debondings and their subsequent coalescence. In the third stage, these micro-debondings are uniformly distributed across the FGA, completing the formation of a penny-shaped crack around the interior defect. Throughout this stage, which comprises more than 95% of the fatigue life, the crack grows at a very slow rate, resulting in a notably rough fracture surface. Once the FGA zone reaches a critical size, the crack initiates accelerated growth in accordance with Paris's law, leading to a significant change in the fracture surface characteristics. The wide D(H) spectrum in the roughness scaling reflects the presence of two self-similar solutions describing these stages of coherent slip with solitary wave dynamics and following blow-up dynamics of damage localization. The universality of D(H) spectrum for HCF and VHCF regimes at the final stage of failure with “monofractality signs” follows to the intermediate self-similar solution for the stress distribution at the crack tip (the Irvin solution)^40^, when the crack length, as the sum of crack origin length and the length of FGA area, is approaching to the Griffith length.

## Conclusion

A comparative analysis of fracture mechanisms in HCF and VHCF regimes was carried out based on the results of multifractal analysis of the fracture surfaces of 316L stainless steel samples produced by laser powder bed fusion. The sets of spectra were constructed by the MF-DFA method from profiles inside the crack nucleation region and its propagation region. The fracture surfaces of samples in the VHCF regime are characterized by the variety of the scaling properties of the fracture relief patterns. The scaling characteristics were classified according to the signs of criticality of stages of damage-failure transition and corresponding self-similar solution for damage kinetics and crack advance. The presence of different self-similar solutions (the attractor types) for characteristic stages allows us to propose the explanation of the nature of qualitative difference of damage-failure transition stages in HCF and VHCF regimes.

## Data Availability

The data generated or analyzed during this study are available from the corresponding author on reasonable request.
